# Removal of Paracetamol from Aqueous Solutions by Photocatalytic Ozonation over TiO_2_-Me_x_O_y_ Thin Films

**DOI:** 10.3390/nano12040613

**Published:** 2022-02-11

**Authors:** Sorin Marius Avramescu, Irina Fierascu, Radu Claudiu Fierascu, Roxana Ioana Brazdis, Angel Vasile Nica, Claudia Butean, Elena Alina Olaru, Sorin Ulinici, Marian Nicolae Verziu, Anca Dumitru

**Affiliations:** 1 Department of Organic Chemistry, Biochemistry and Catalysis, Faculty of Chemistry, University of Bucharest, 050663 Bucharest, Romania; sorin.avramescu@g.unibuc.ro; 2 PROTMED Research Centre, University of Bucharest, 050107 Bucharest, Romania; nica.angelvasile@yahoo.ro (A.V.N.); alina.olaru@g.unibuc.ro (E.A.O.); 3 Emerging Nanotechnologies Group, National Institute for Research & Development in Chemistry and Petrochemistry—ICECHIM, 060021 Bucharest, Romania; roxana.brazdis@icechim.ro; 4 Faculty of Horticulture, University of Agronomic Sciences and Veterinary Medicine of Bucharest, 011464 Bucharest, Romania; 5 Department of Science and Engineering of Oxide Materials and Nanomaterials, Faculty of Chemical Engineering and Biotechnologies, University Politehnica of Bucharest, 011061 Bucharest, Romania; 6 Department of Chemistry and Biology, North University Centre of Baia Mare, Technical University of Cluj-Napoca, 430122 Baia Mare, Romania; dee1168@yahoo.com; 7 S.C. ICPE Bistrita S.A, 420035 Bistrita, Romania; sorin_ulinici@icpebn.ro; 8 Department of Bioresources and Polymer Science, Advanced Polymer Materials Group, Faculty of Chemical Engineering and Biotechnologies, University Politehnica of Bucharest, 1-7 Gh Polizu Street, 011061 Bucharest, Romania; marian.verziu@upb.ro; 9 Faculty of Physics, University of Bucharest, 077125 Magurele, Romania

**Keywords:** catalytic ozonation, water treatment, paracetamol, photo-catalysis, thin films, titanium dioxide

## Abstract

Analgesics and nonsteroidal anti-inflammatory drugs (NSAIDs) such as paracetamol, diclofenac, and ibuprofen are frequently encountered in surface and ground water, thereby posing a significant risk to aquatic ecosystems. Our study reports the catalytic performances of nanosystems TiO_2_-Me_x_O_y_ (Me = Ce, Sn) prepared by the sol-gel method and deposited onto glass slides by a dip-coating approach in the removal of paracetamol from aqueous solutions by catalytic ozonation. The effect of catalyst type and operation parameters on oxidation efficiency was assessed. In addition to improving this process, the present work simplifies it by avoiding the difficult step of catalyst separation. It was found that the thin films were capable of removing all pollutants from target compounds to the oxidation products.

## 1. Introduction

Water supplies are under a constant threat due to pollution resulting from numerous and complex anthropic activities. A special situation is represented by pharmaceuticals which escape traditional water treatment methods and reach the water bodies from multiple and almost uncontrollable sources [1,2,3,4]: effluents from pharmaceutical industries and hospitals, household disposal, the excretion of non-metabolized/metabolized drugs after human/animal consumption, aquaculture, sludge from water treatment facilities used as fertilizer and leaching drugs in ground water, etc. Moreover, along with parent compounds, the metabolites can also be harmful to aquatic ecosystems. The fraction of medicines that remain unchanged and leave patients’ bodies ranges between 25% and 75% and, considering the large number of pharmaceuticals used worldwide, this results in a significant environmental burden which is growing due to an increase in world population in number and age. Thus, in 2020, 4.5 trillion doses of different drugs were used and 50% of the world’s population consume more than one dose [5] and, consequently, the concentration of pharmaceuticals in numerous natural water sources increase dramatically [6]. These compounds are encompassed (along with other largely used chemical compounds, such as hormones, plasticizers, wood treatment, pesticides, etc.) in the category of emerging pollutants (Eps) which are contaminants that are not currently regulated by environmental agencies. Another aspect of concern is that most of these compounds are also circumvented to the endocrine disruptor category that increases the danger for aquatic ecosystems.

Analgesics and nonsteroidal anti-inflammatory drugs (NSAIDs) like paracetamol, diclofenac, and ibuprofen are the most frequently encountered in surface and ground water, and therefore represent a significant risk for aquatic ecosystems [7,8]. Among them, paracetamol has an over-the-counter regime and is used intensively for pain relief and fever reduction. It is produced in staggering amounts, and the global acetaminophen market was valued at around US 850.5 million in 2020 and is expected to reach US 977.5 million in 2027, with a compound annual growth rate (CAGR) of around 2% [9]. In some countries, paracetamol consumption exceeds 20 g/person every year [10], thus it ends up in surface waters at concentrations of around 100 μg/L and surpasses tenfold the predicted non-effect concentration parameter (PNEC) which is 9.2 μg/L. Hence, considering the huge environmental impact of this compound and its metabolites, it is important to reduce the spreading of this drug in different aquatic ecosystems. In fact, it is primarily a containment problem and, as to solve this issue, paracetamol leaking must be stopped, preferably at the source. In order to fulfill this difficult task, it is necessary to use a versatile and efficient water treatment method, with large applicability in terms of aqueous effluents flow rate, source type, and the removal of target pollutants at the level requested by environmental regulation (which are subject to change toward lower values in a rapid pace). Catalytic ozonation is a technique with a significant capacity to accomplish these requirements in every respect due to the capability to oxidize/mineralize almost every organic pollutant [11,12,13,14,15,16,17,18,19,20,21]. Moreover, this method is a prominent example of advanced oxidation processes—a well-known category of water treatment approaches centered on using OH● radicals. This is the reason for a large number of studies regarding the development of catalytic systems capable of assisting the oxidative power of ozone mainly through the radical route. A large number of solids based on transitional metals in complex form or oxides, carbonaceous materials, etc., were studied for an evaluation of catalytic activity [22,23,24,25,26,27,28,29,30]. Additionally, catalyst precursors can be obtained from different industrial or agricultural wastes or pure metals salts. Despite that the catalytic ozonation process relies on a complex heterogeneous system (solid-gas-liquid), its performances are remarkable since most of these systems eliminate recalcitrant pollutants and set the quality parameters of treated water in the range established by environmental agencies. To be able to avoid the cumbersome separation step of the catalyst from treated water, a catalyst with a magnetic core was introduced [23,31,32,33,34,35,36]. Another interesting approach, that is unexploited in catalytic ozonation, although widely used in various other applications, is represented by thin films [37,38,39,40,41,42,43,44].

Well-known for its catalytic properties and chemical stability, TiO_2_ represents a very good candidate for the development of new nanosystems with an environmental application, however, its photocatalytic applications are limited, as a consequence of the large band gap (~3.2 eV) and fast recombination of the exciton of TiO_2_. This drawback can be overcome by the use of heterostructures of TiO_2_ and metal oxides with lower valence bands (i.e., SnO_2_), which hinders charge recombination, thus obtaining efficient photo-catalysis [45]. In an attempt to overcome another drawback in applying photo-catalysis to the treatment of polluted water (the recovery of the powder catalyst), TiO_2_-based thin films, immobilized on different supports (aerogels, polymers, glass, etc.) were successfully proposed in the literature [46,47].

In this study, we report for the first time the removal of paracetamol from aqueous solutions using thin film catalysts based on TiO_2_-Me_x_O_y_ nanosystems deposited on glass plates and immersed into a semi-batch reactor. The influence of catalyst type and operational parameters in oxidation efficiency was assessed.

## 2. Materials and Methods

### 2.1. Chemicals and Materials

Commercially available reagents with a purity higher than 98% for solid compounds and an HPLC grade for solvents were purchased from Sigma–Aldrich (Baden-Württemberg, Germany): paracetamol, Triton X-100 (X100, polyethylene glycol tert-octyl phenyl ether), titanium tetraisopropoxide (TTIP), tin (IV) chloride pentahydrate, cerium (III) nitrate hexahydrate, acetic acid (AcOH), ethanol (EtOH), acetonitrile (MeCN) and trifluoracetic acid (TFA). Borosilicate glass slides (25 × 75 mm and a thickness of 1.0–1.2 mm) were obtained from Labbox (Barcelona, Spain). Airflow for ozone generation was provided by a laboratory compressor and distilled water with a resistivity of 18.2 MΩ.cm was used in all experiments.

### 2.2. Sol-Gel Synthesis

In sol-gel synthesis usually, a pore directing agent is used and Triton X100 (a nonionic surfactant with long chain) was selected for this study. Triton X100 is an inexpensive and nontoxic amphiphilic molecule, with a significant capacity to accommodate inorganic structures in aqueous liquids. A solution of ethanol, Triton X100, and acetic acid were prepared and, in this mixture, TTIP was introduced under vigorous stirring. The molar ratio of these components (X100:EtOH:AcOH:TTIP = 1:70:5:1) was selected in order to achieve a higher specific surface based on optimization studies undertaken by Stathatos et al. [48]. Acetic acid and ethanol release water upon esterification and consequently, slow hydrolysis of TTIP take effect. Additionally, the acetic acid replaces, to some extent, the alkoxy groups in TTIP. Each mentioned metals salt was added to separate flasks containing the above-prepared solution in quantities, so Me is 5% of the TTIP content except for two metal modified thin films, where the percent of metal attain a cumulative 10%.

### 2.3. Preparation of Thin Films

Glass slides were cleaned thoroughly in a succession of wash stages: detergent, water, acetone, and drying in N_2_ flow. Thin films were deposited on slides using the dip-coating method using a programmable system (80C—PTL-SC-6-LD, MTI, New York, NY, USA) following several steps: immersion and extraction of slides from solution at a rate of 10 cm/min for three times with a 30 min pause between the dipping operation for drying at 60 °C. Finally, each slide was calcined in a multistep programmable furnace (Nabertherm L5/11, Neuhausen, Germany) at a ramp rate of 5 °C/min to 550 °C, maintained at 550 °C for 90 min and cooled down naturally. Prepared films on glass slides will be referred to as (Ti-Me) or (Ti-Me1-Me2) where Me = Ce, Sn.

### 2.4. Thin Films Characterization

The thin films’ characterization was realized through several methods. The XRD patterns were recorded using Bruker D8 Discover equipment (Bruker AXS, Karlsruhe, Germany) (Cu_kα1,_ λ = 1.5406 Å), between 15 and 70° (2θ), in a grazing incidence configuration. The topography of the coated thin films was analyzed using an A.P.E Research SPM atomic force microscope (A.P.E. Research SRL, Trieste, Italy). DRIFT spectra of samples were collected in the region of 4000–500 cm^−1^ at a resolution of 2 cm^−1^ on a Varian 3100 Excalibur spectrometer (Walnut Creek, CA, USA) equipped with a Harrick Praying Mantis (Harrick Scientific Products, Inc., Pleasantville, NY, USA) diffuse reflectance accessory. UV–Vis absorption spectra of the films were carried out with a Helios alpha (Unicam, Cambridge, UK) spectrometer. The optical band gap [49,50,51] was evaluated by using the Tauc and Davis–Mott relation:

(1) $$\alpha h\upsilon =A{\left(h\upsilon -E_{g}\right)}^{n}$$

where *α* is the absorption coefficient, *A* is an energy-independent constant, *hυ* stands for photon energy, *Eg* is the optical energy band gap, and *n* is considered to be the transition fingerprint. For *n* = 1/2, 3/2, 2, and 3, it can be distinguish direct allowed, direct forbidden, indirect allowed, and indirect forbidden transitions, respectively. Tauc graphs were developed by plotting *αhυ*^n^ as a function of energy (*hυ*), and the point where the tangent of the reverse-extension curve intersects with the *x*-axis represents the band gap value of a semiconductor [49].

Raman spectra were carried out using a Renishaw in Via Confocal Raman (Renishaw, Wotton-under-Edge, Gloucestershire, UK) microscope system. The excitation laser wavelength was 473 nm. The Raman spectra were acquired in the extended spectral region from 100 to 3200 cm^−1^, under ambient conditions.

### 2.5. Photoreactor, Photocatalytic Activity, and Aqueous Effluents Analysis

**Figure 1:**
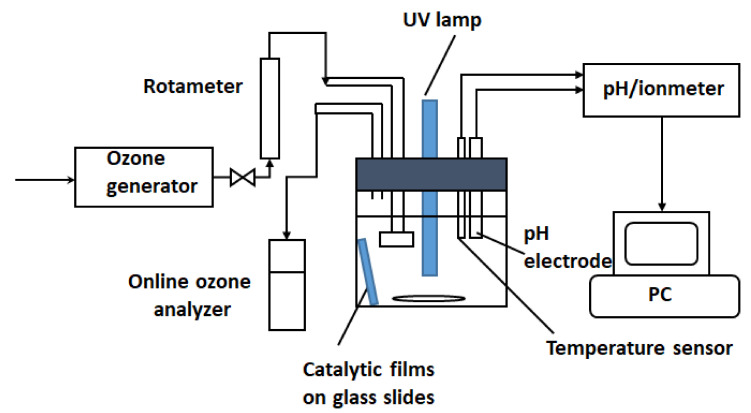
Experimental set-up for photocatalytic ozonation processes.

All experiments regarding oxidation of paracetamol were performed in a semi-batch jacketed cylindrical reactor with a capacity of 250 mL equipped with a Pen-Ray UV Light Source 254 nm (Cole-Parmer, Vernon Hills, IL, USA) protected by a quartz tube and placed centrally (Figure 1). Ozone was obtained from dried air using an ozone generator (COM-AD-01, Anseros, Tübingen, Germany) and gas-phase ozone concentration was determined with an ozone analyzer (BMT 964, Stahnsdorf, Germany). The gas flow was supplied through a gas inlet into the reaction solution while simultaneously stirring at 1000 rpm, the temperature was kept at 20 °C using a thermostat, and solution pH was measured (PHM 240, Radiometer S.A.S., Neuilly-Plaisance, France) by an electrode inserted directly into the solution and maintained at 7 by adding HCl or NaOH trough liquid inlets. Samples were withdrawn at certain time intervals (5 to 30 min) to assure complete monitoring of the reaction course. The whole system was covered with aluminum foil as a deflector. Two or four glass slides with catalyst deposited on them were accommodated inside the reactor during the reaction. The reactor was filled with a 200 mL aqueous solution of paracetamol (100 mg/L). For repeated use, the catalytic slides were washed with distilled water and dried at 100 °C without any other treatment.

Paracetamol concentration was determined by the HPLC method using an L-3000 system (Rigol Technologies Inc., Beijing, China) consisting of a quaternary pump, a diode-array detector, and a Kinetex C18 Evo (150 mm × 4.6 mm i.d.; 5 μm particle size; Phenomenex, Torrance, CA, USA). Operating conditions were: isocratic elution of the mobile phase composed of eluent A (0.1% TFA in acetonitrile); eluent B (0.1% TFA in water) = 20:80, flow rate 1.0 mL/min, injection volume 10 μL, detection at 220 nm and 30 °C. Ammonia and nitrate ions were determined using a Varian HPLC system ProStar (Varian Analytical Instruments, Palo Alto, CA, USA) equipped with a conductometric detector, a conductivity suppressor, and Dionex cation or anion columns (IONPAC AS 19—4 × 250 mm or IonPac CS19—4 × 250 mm, Thermo Scientific, Waltham, MA. USA). Analysis was performed in isocratic mode with buffer carbonate/bicarbonate for nitrate and methanesulphonic acid for ammonia. Total organic carbon (TOC) measurements were achieved by the high-temperature oxidation method using a HiPerTOC (Thermo Electron, Waltham, MA, USA) analyzer. The absorbance of water samples at 254 nm (UV254) was measured using a Helios alpha (Unicam, Cambridge, UK) spectrometer.

## 3. Results and Discussions

### 3.1. Thin Film Characterization

**Figure 2:**
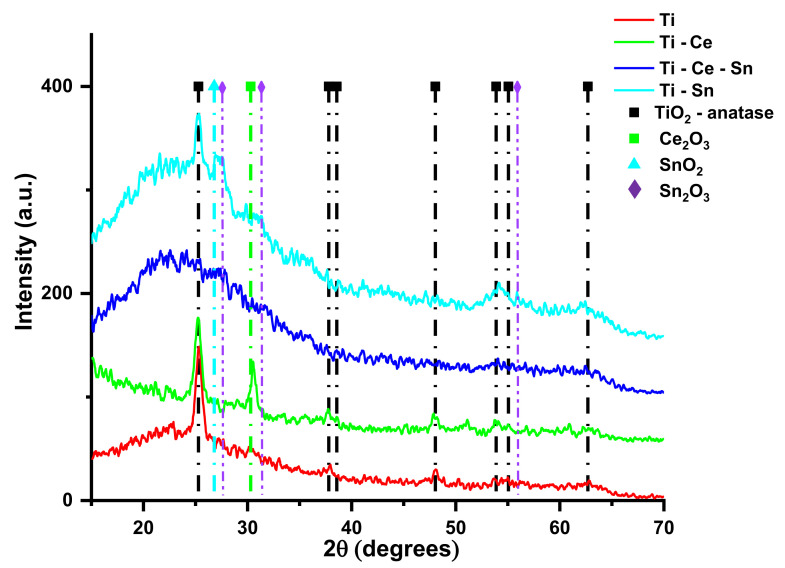
XRD patterns of TiO_2_-Me_x_O_y_ films.

The XRD patterns of the obtained materials are shown in Figure 2. The diffractograms of the as-prepared TiO_2_ film reveal a typical anatase structure (according to pdf file 00-064-0863 from the International Centre of Diffraction Data 2019), with the most intense (101) peak at 2θ = 25.08° [52,53] and crystallite size of approximately 11.5 nm, calculated using the Debye–Scherrer equation using the (101) peak. The diffractograms also presents other peaks, corresponding to the diffraction planes (004), (112), (200), (105), (211), and (204) at 2θ = 37.7, 38.6, 48.1, 53.9, 55.1, respectively, 62.7°. The XRD pattern for as-prepared (Ti-Ce) shows an additional peak compared with TiO_2_ films at around 30.3°, which is attributed to the Ce_2_O_3_ (101) plane according to pdf file 00-023-1048 from the International Centre of Diffraction Data 2019 [54]. According to literature data, the most probable explanation is related, on one hand, to a poor crystallization of the cerium oxide phase, which would not allow the indexation of other peaks, and on the other hand, to the low concentration of the phase, compared with the TiO_2_ support [55,56,57]. Other authors [57] identified the presence of both CeO_2_ and Ce_2_O_3_ phases in the XRD data; however, in our obtained diffractograms, no discernable peaks that can be attributed to a CeO_2_ phase could be identified. The crystallite size of the TiO_2_ phase was calculated to be approximately 12 nm, while the crystallite size of the the Ce_2_O_3_ phase was approximately 19 nm. The main conclusion is that Ce_2_O_3_ has little effect on the crystal structure of TiO_2_. The XRD pattern of (Ti-Sn) films shows that the diffraction peaks corresponding to the anatase phase are slightly wider, with a crystallite size of approximately 13 nm. Additionally, the diffractogram reveals the presence of the specific amorphous phase peak, around 25° (2θ). Moreover, an additional contribution in the diffraction pattern of (Ti-Sn) films which are attributed to the tetragonal SnO_2_ plane (110) can be observed at 2θ = 26.6°, in concordance with pdf file data from the International Centre of Diffraction Data 2019 (00-005-0467), with a crystallite size corresponding to the SnO_2_ phase over 20 nm. Several other minor peaks are present in the Ti-Sn diffractogram, which can be attributed to the presence of secondary phase, most probably Sn_2_O_3_ (ICDD PDF card no. 00-025-1259), as suggested by the presence of minor diffraction peaks at 2θ = 27.4, 31.4, and 55.9°, corresponding to the diffraction planes (011), (021), respectively (141). The diffraction pattern of as-prepared Ti-Ce-Sn does not show any diffraction peaks except an overlap contribution of around 25° (2θ), which indicates an amorphous nature of the thin film. Similar amorphous films were also reported by other authors, at a relatively Ti/Me ratio [58].

**Figure 3:**
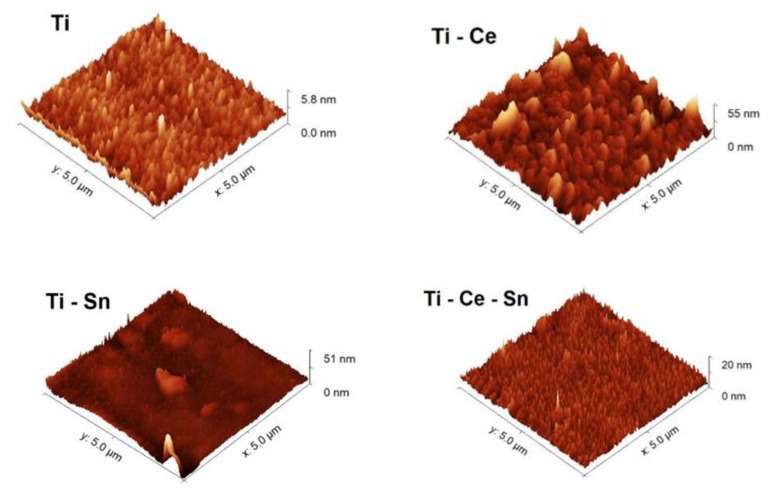
AFM images of TiO_2_-Me_x_O_y_ films.

For the prepared thin films, AFM images and surface data are presented in Figure 3 and Table 1, showing that all modified TiO_2_-Me films were rougher than the simple TiO_2_ films. In terms of catalytic activity, increased surface irregularities are subject to providing more active sites therefore faster removal of pollutants.

**Figure 4:**
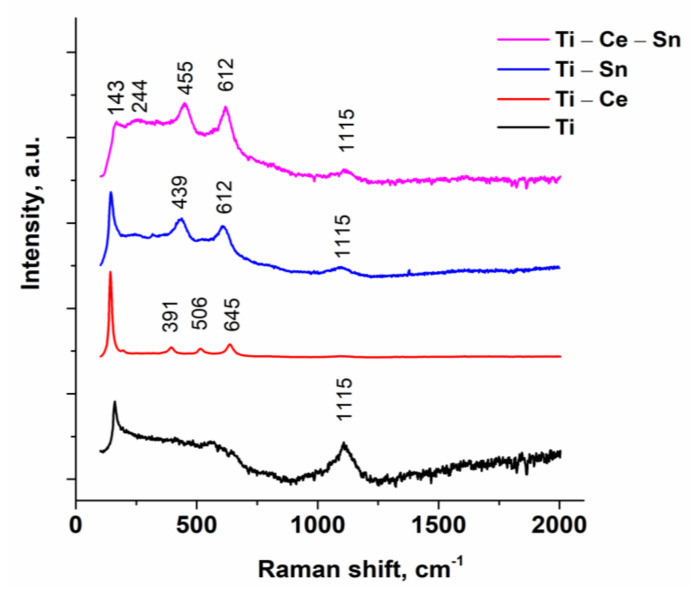
Raman spectra of prepared film.

Raman spectroscopy is a widely used tool to detect chemical bonds as well as to identify the crystalline state of metal oxides. Raman spectra (Figure 4) for all prepared films were performed under ambient conditions. The band at 143 cm^−1^ assigned to TiO_2_ anatase structure [59] is well highlighted for all materials, except for the Ti-Ce-Sn sample, where the presence both of cerium and tin led to an increase of bands intensity at 244, 455, and 612 cm^−1^ which corresponds to the rutile phase [60]. Moreover, SnO_2_ promotes the crystallization of the TiO_2_ in the rutile phase [61,62], which justifies the presence of the bands’ characteristics for rutile structure regarding the Ti-Sn films. On the other hand, in the presence of Ce, the Raman bands are below 700 cm^−1^ and could be assigned to TiO_2_ as anatase [63].

Anatase presence is consistent with data obtained from XRD patterns. In the case of the (Ti-Ce) system, three bands at 391, 506 and 645 cm^−1^ confirm the formation of crystalline CeO_2_ [64,65]. The spectra for (Ti-Sn) systems present bands at 442 cm^−1^ with a shift at 455 cm^−1^ for (Ti-Ce-Sn), a result of Eg vibration mode for the rutile phase of SnO_2_ and at 612 cm^−1^ for both systems attributed surface phonon modes of SnO_2_ [66]. For all spectra, the band at 1115 cm^−1^ corresponds to a glass substrate.

FTIR spectra (Figure S1, Supplementary Material) were collected for pristine films and those used in the oxidation reaction. Borosilicate glass slides present a characteristic band assigned to Si–O bonds (559, 1287 cm^−1^), Bi–O (826, 2162 cm^−1^), OH groups (3532 cm^−1^), and the spectra for (Ti) and (Ti-Ce) films mimics glass spectra, probably due to higher transparency. In the case of (T-Sn) and (Ti-Ce-Sn) systems, FTIR spectra are significantly flattened as the thickness increases. FTIR spectra were also used as a tool for assessing the preservation of film integrity during the oxidation process and it can be observed that the thin films preserve their integrity.

**Figure 5:**
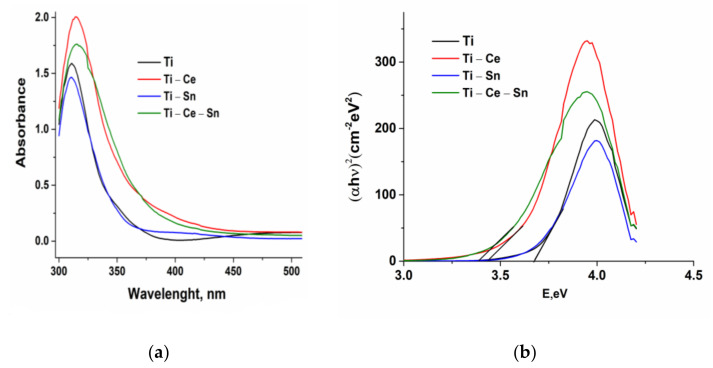
Absorbance data (**a**) and Tauc plot (**b**) for prepared films.

The UV-VIS absorption spectra and Tauc plots of modified TiO_2_ thin films were presented in Figure 5 and the estimated band gaps (*Eg*) are presented in Table 2. Lower values of E_g_ for Ce modified films can be attributed to partial substitution of Ti cations and/or TiO_2_ crystal size [67]. From the transmittance spectra, it can be observed that prepared films are highly transparent in the visible region with a red shift of (Ti-Ce), (Ti-Ce-Sn) compared with (Ti) and (Ti-Sn) and this is an indication that these films have a narrow bandwidth [65]. Increased transmission is also linked with film crystallinity [68] and consequently, to photocatalytic activity.

### 3.2. Photocatalytic Ozonation Tests

**Figure 6:**
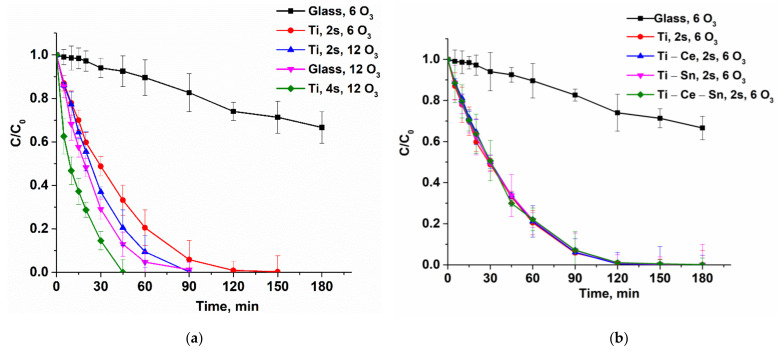
(**a**) Evolution of paracetamol normalized concentration during the oxidation process for (Ti) system at different ozone concentrations and number of slides; (**b**) Comparison of prepared catalytic systems at a lower ozone input and two slides.

**Figure 7:**
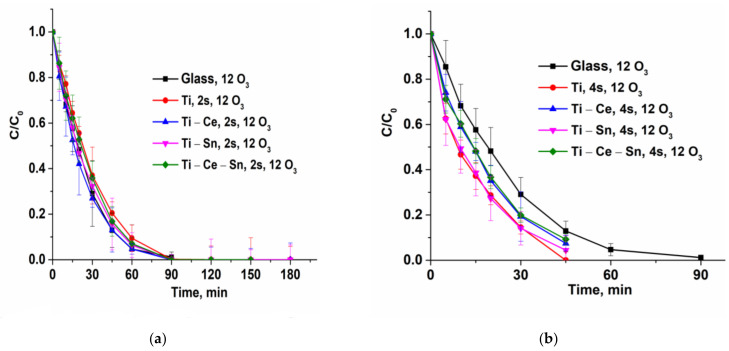
(**a**) Comparison of prepared catalytic systems at a higher ozone input and two slides; (**b**) Comparison of prepared catalytic systems at a higher ozone input and four slides.

**Figure 8:**
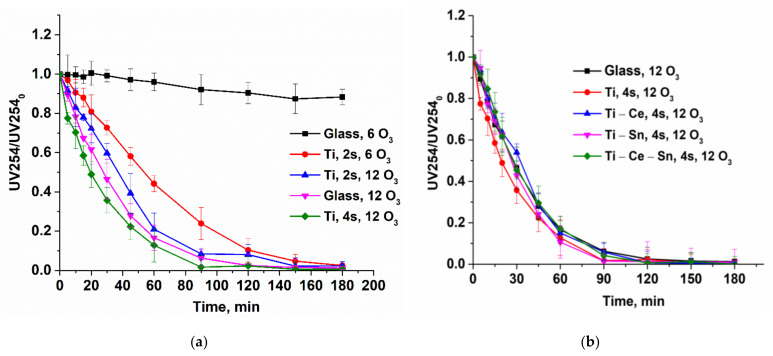
(**a**) Evolution of normalized UV254 parameter for (Ti) system in different reaction conditions; (**b**) Evolution of a normalized UV254 parameter for all prepared systems at a higher ozone input and four slides.

**Figure 9:**
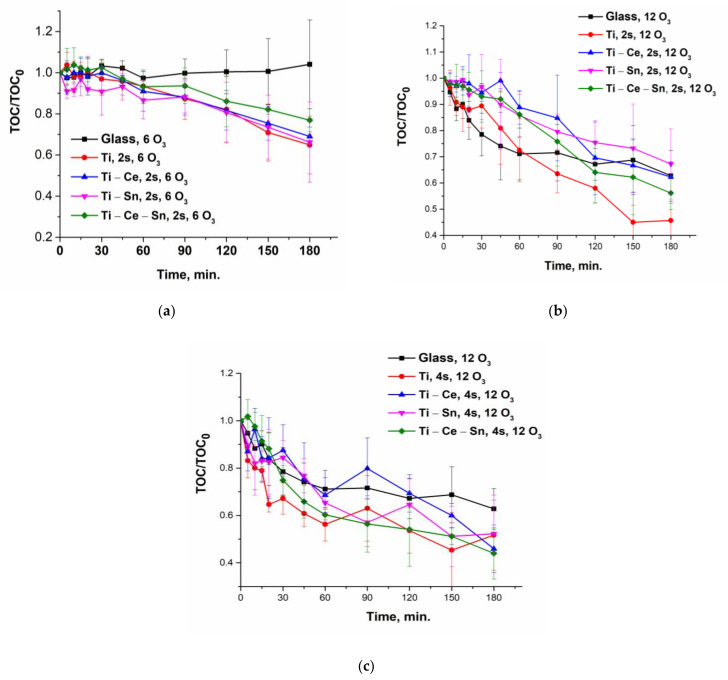
(**a**) Evolution of normalized TOC for all prepared systems at a lower ozone input and two slides; (**b**) Evolution of normalized TOC for all prepared systems at a higher ozone input and two slides; (**c**) Evolution of normalized TOC for all prepared systems at a higher ozone input and four slides.

Catalytic tests were performed for all prepared films using the following operational parameters: pH = 7, gas flow rate = 10 L/h, temperature = 20 °C, two or four catalytic slides, and two different O_3_ concentrations: 6 or 12 g/Nm^3^ (equivalent to 1 mg/min and 2 mg/min, respectively). For noncatalytic tests, bare glass slides were used. The reactions were repeated once with the same slides for verification of catalysts’ reusability and, for all runs, a multiparametric assessment of the process was performed. Thus, several chemical species and parameters were monitored: paracetamol concentration, total organic carbon (TOC), solution absorbance at 254 nm, ammonia ions, and nitrate ions. From the evolution of paracetamol normalized concentrations (Figure 6) it can be observed that for lower ozone input (6 g/Nm^3^), the noncatalytic process occurs very slow compared with catalytic ones. With the increasing O_3_ concentration (12 g/Nm^3^), the reaction rate for the noncatalytic process rose significantly and became comparable with catalytic tests performed with two catalytic slides (Figure 7a). However, when using four slides (Figure 7b), the oxidation of the pollutant occurs much more rapidly and the target compound is removed in less than 45 min for (Ti) and (Ti-Sn) systems and in nearly 90 min in absence of catalysts. The reusability of catalytic slides is proved for all systems by the same evolution of normalized paracetamol concentration for two consecutive reactions (Figures S2–S5, Supplementary Material). Monitoring the process only from the target pollutant perspective is not a good choice since the oxidation byproducts can also be toxic and require advanced removal. Thus, UV absorbance at 254 nm (UV254) for prelevated samples during the reactions is generally considered as an indicator of the presence of unsaturated carbon bonds (mostly from aromatic compounds) and is a useful monitoring tool [69,70,71,72,73,74,75,76]. Figure 8 shows that the evolution of the aromatic and/or double bond containing oxidation byproducts follows the same behavior such as the removal of the parent compound and is persistent throughout the entire reactions, especially for processes with a low ozone concentration of two catalytic slides. The reason for this is that the paracetamol molecule loses the acetamide group which is further transformed into ammonia ions. Moreover, the core of the molecule is more or less destroyed (depending on reaction conditions) by consecutive augmentation with oxygen, followed by cycle opening and subsequent transformation in smaller moieties [77,78,79,80,81]. Additionally, this parameter was used to verify the reusability of the catalytic system and it was found that for two consecutive reactions the activity remains unchanged (Figures S6–S9, Supplementary Material). The gradual destruction of pollutant molecules is the reason for the slow mineralization process, as can be observed from Figure 9a–c where TOC/TOC_o_ evolution versus time is depicted. The elimination of organic carbon by mineralization is almost absent without catalysts at 6 g O_3_/Nm^3^ m and, in the presence of a catalyst, the TOC removal attains 35%. Doubling the ozone dose and using two catalyst slides lead to TOC elimination and up to 55% for (Ti) and 48% for (Ti-Ce-Sn) thin films, while for noncatalytic reaction, mineralization occurs up to 38%. By using four catalytic slides, mineralization is improved up to ~68% for all systems.

**Figure 10:**
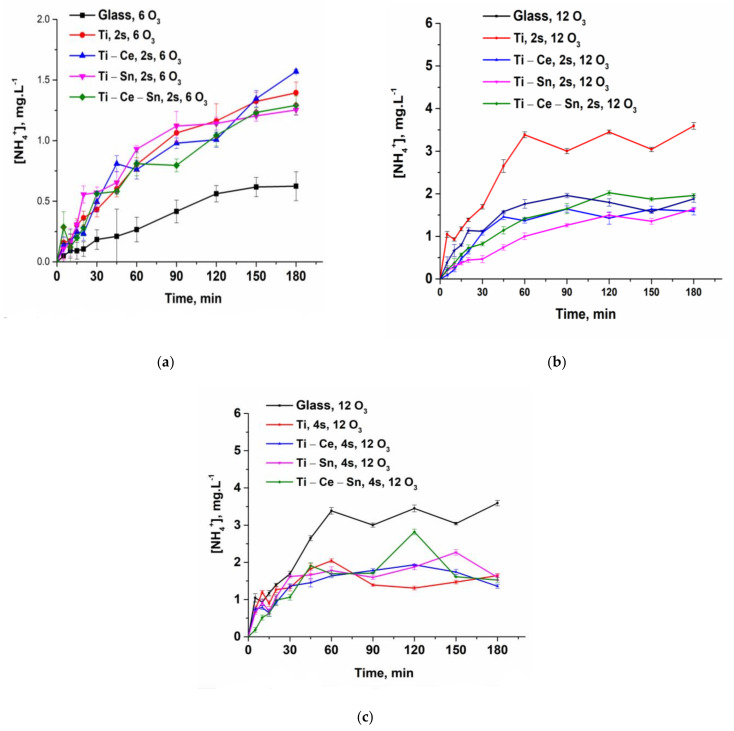
(**a**) Evolution of ammonia ions for all prepared systems at a lower ozone input and two slides; (**b**) Evolution of ammonia ions for all prepared systems at a higher ozone input and two slides; (**c**) Evolution of ammonia ions for all prepared systems at a higher ozone input and four slides.

**Figure 11:**
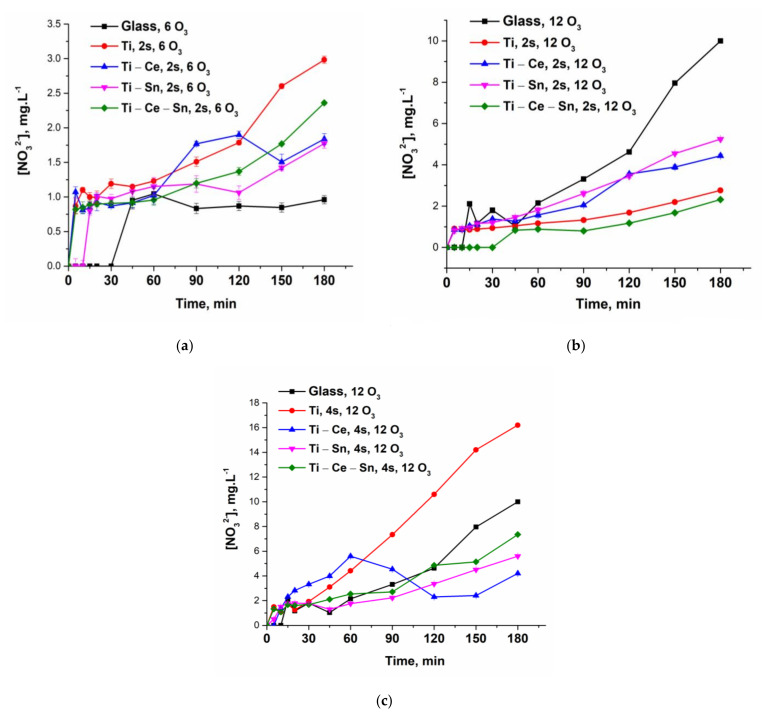
(**a**) Evolution of nitrate ions for all prepared systems at a lower ozone input and two slides; (**b**) Evolution of nitrate ions for all prepared systems at a higher ozone input and two slides; (**c**) Evolution of nitrate ions for all prepared systems at a higher ozone input and four slides.

During the oxidation process besides the different organic molecules, another toxic and refractory pollutant is accumulated in the reaction system, namely, ammonia. Ammonia removal represents, in general, a significant challenge due to strict environmental regulations and, the main elimination route is by biological processes which require some demanding conditions to be applied (high capital costs, difficult process monitoring, large to medium effluents volumes, etc.) [82,83]. Catalytic ozonation, due to its flexibility as a process, can overcome these issues and eliminate the noxious ions. The only concern is the formation of nitrate ions but, in this case, the maximum concentration limit is significantly higher than those for ammonia. Hence, from Figure 10a–c presenting the ammonia build-up and subsequent removal, and Figure 11a–c, which shows the formation of nitrate ions, it can be observed that the presence of prepared catalytic systems can eliminate the toxic ions. Thus, for a lower ozone concentration, the amount of ammonia released during the noncatalytic test is much smaller compared to all catalytic systems since the overall process is very slow (not only for ammonia). By doubling the ozone concentration and in absence of a catalyst, the outcome is a higher accumulation of ammonia until some point where the concentration reaches a plateau. On the contrary, for prepared thin films regardless of the number of slides used and ozone concentration, the ammonia is oxidized at a rapid pace, as can be detected from the nitrate formation. However, the most active systems for ammonia removal are (Ti) and (Ti-Ce-Sn).

#### 3.2.1. Ozone Consumption

**Figure 12:**
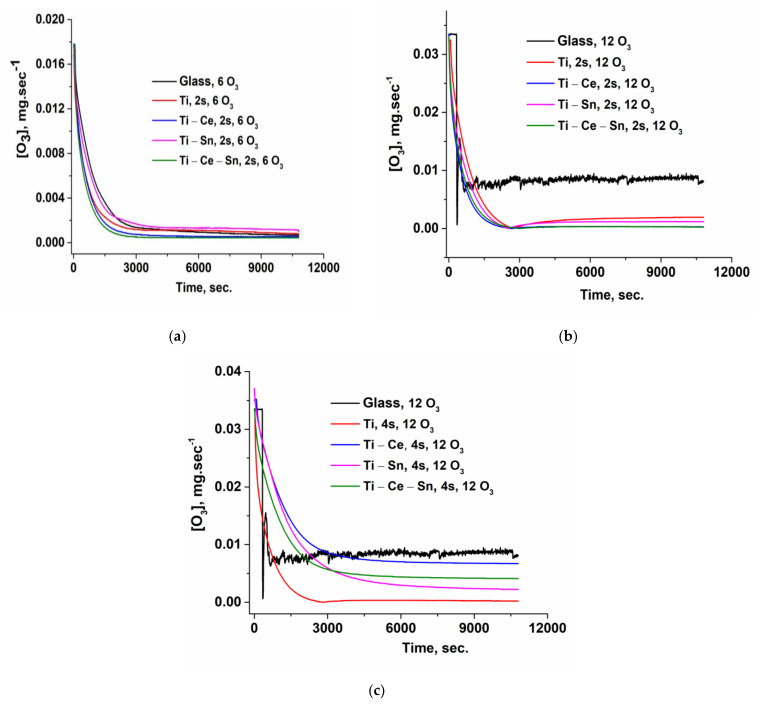
(**a**) Ozone concentration evolution in time for a lower ozone input and two slides; (**b**) Ozone concentration evolution in time for a higher ozone input and two slides; (**c**) Ozone concentration evolution in time for a higher ozone input and four slides.

Ozone consumption is an important parameter that governs the efficiency of the process from an economical perspective. Using an online analyzer is a much easier and accurate method to obtain the real ozone consumption compared with the iodometric approach. During a reaction, the amount of ozone that enters the reactor is not entirely consumed and, the residual ozone is lost without the possibility of being recovered. Hence, the main objectives in the oxidation processes are using in large amounts the reactants, and completely removing the organic matter. From Figure 12a–c and Table 3, it can be observed that ozone is efficiently used in the presence of catalysts, even for lower input concentrations of the oxidant agent compared with not-catalytic processes. These values were obtained by integrating the area below the ozone evolution curve using Origin lab software. Additionally, it can be noticed that in terms of TOC removal, (Ti-Ce) and (Ti-Ce-Sn) are the most effective systems with 36.33 and 39.53 mg O_3_/mg TOC (for 4 slides) or 15.21 and 12.72 mg O_3_/mg TOC removed (for 2 slides).

#### 3.2.2. Kinetic Study

**Figure 13:**
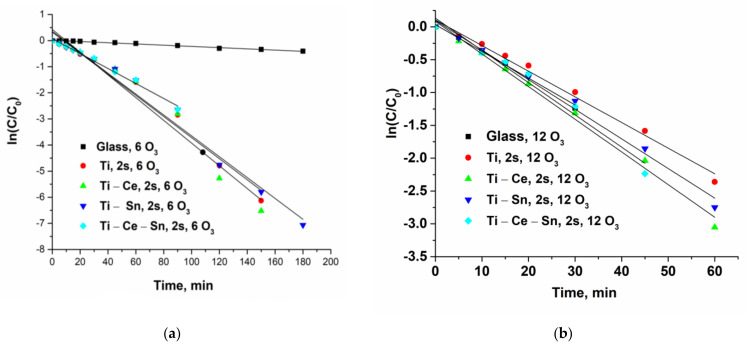

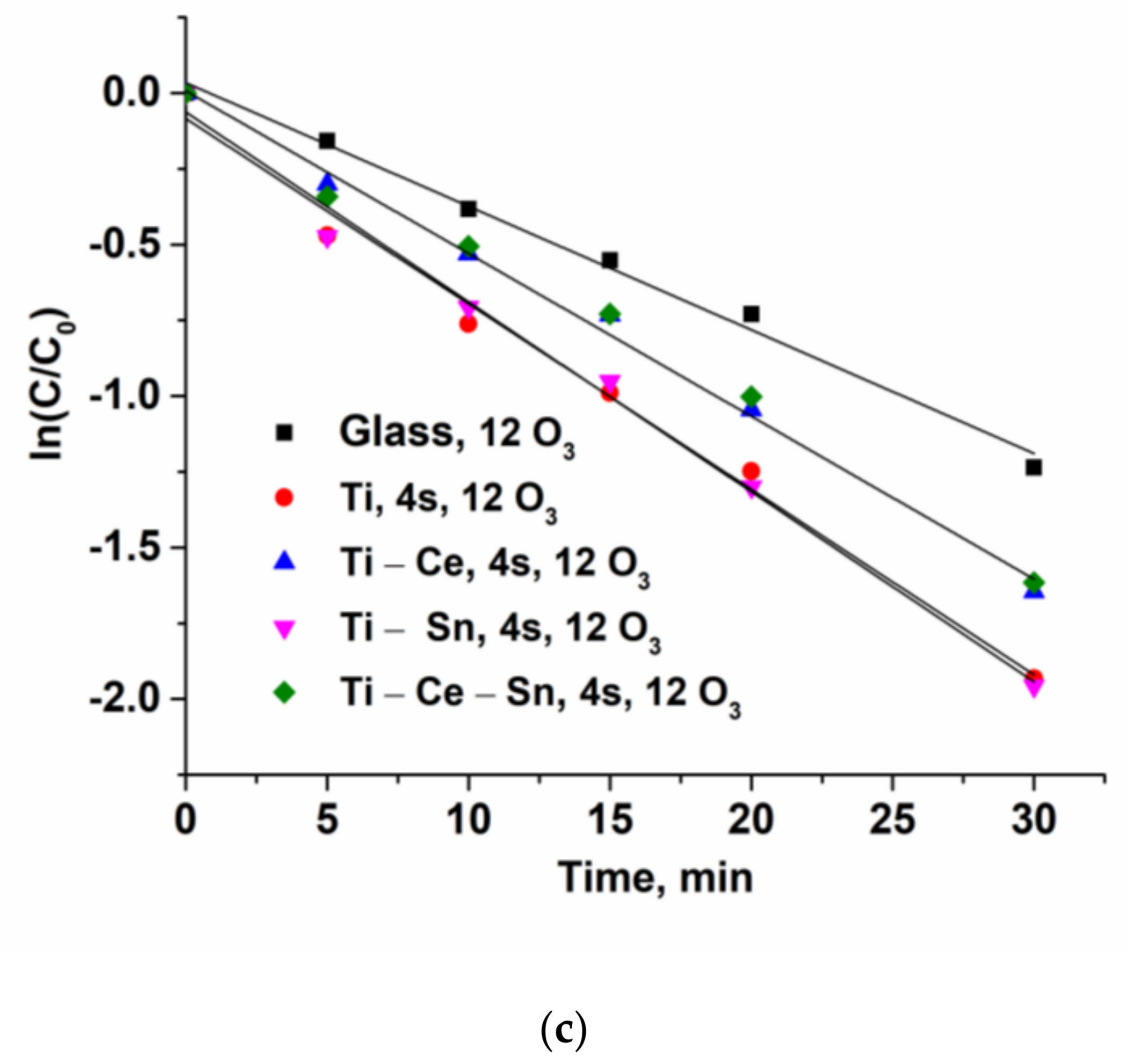
(**a**) Pseudo-first-order plots of ln(C/C_o_) lower ozone input and two slides; (**b**) Pseudo-first-order plots of ln(C/C_o_) higher ozone input and two slides; (**c**) Pseudo-first-order plots of ln(C/C_o_) higher ozone input and four slides.

Paracetamol (*Pa*) ozonation in the presence and absence of catalysts can be modeled by a pseudo-first-order kinetic (Equations (2)–(4)) where *k*_1_ and *k*_2_ are the reaction rate constants of pollutant interaction with ozone and *OH*● respectively. By plotting ln[*Pa*]/[*Pa*]_o_ versus time (Equation (5), Figure 13a–c), the observed constants (*k*_*obs cat*_ and *k*_*obs not-cat*)_ will be obtained and are presented in Table 4. The values of the kinetic constants are used as indicators for comparing the performances of catalytic and not–catalytic processes. It can be observed that most active systems are (Ti) and (Ti-Ce-Sn) since their removal rate constant of paracetamol are 1.5 and 1.53 times, respectively, higher than that of the noncatalytic process.

(2) $$-\frac{d\left[Pa\right]}{dt}=k_{1}\left[O_{3}\right]\left[Pa\right]+k_{2}\left[OH●\right]\left[Pa\right]=k_{obs\;cat}\left[Pa\right]$$

(3) $$O_{3}+Catalyst\;active\;sites\to OH●$$

(4) $$-\frac{d\left[Pa\right]}{dt}=k_{1}\left[O_{3}\right]\left[Pa\right]=k_{obs\;not-cat}\left[Pa\right]$$

(5) $$ln\left(\frac{\left[Pa\right]}{{\left[Pa\right]}_{0}}\right)=-k_{obs}t$$

#### 3.2.3. Photocatalytic Mechanism

**Figure 14:**
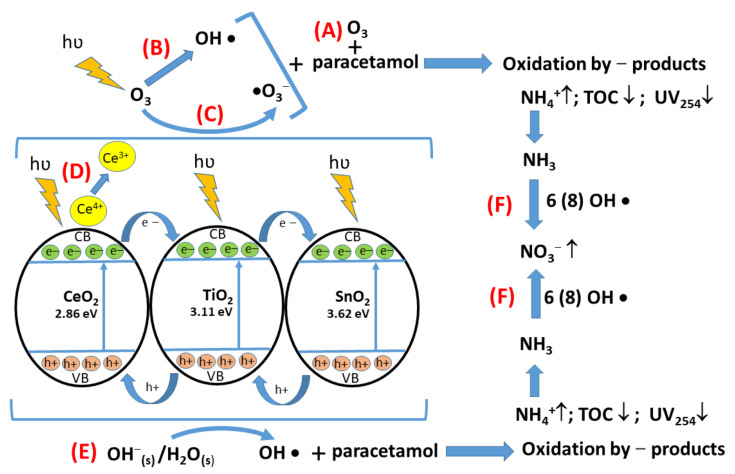
Proposed photocatalytic mechanism for paracetamol oxidation; occurring processes: direct oxidation with ozone (**A**); radical production in the presence of UV radiation (**B**); interactions of formed electrons and holes with O_3_ and OH (**C**,**E**); Ce^3+^/Ce^4+^ formation (**D**), NH_3_ oxidation (**F**).

Photocatalytic ozonation of different pollutants generally involves several steps and routes [24,84,85,86,87,88,89,90]. In this study, from the reactions at small ozone concentration (6O_3_), it can be observed that direct oxidation with ozone (Figure 14A) has low importance (Equations (6) and (7)), while at a higher ozone concentration (12O_3_) and in the presence of UV radiation (Figure 14B), the radical production increases significantly (Equations (8)–(10)). In the presence of catalysts, several processes occur. The process starts with electrons transfer from the valence band to the conduction band as a result of radiation excitation. Thus, the formed electrons and holes are available for interaction with O_3_ and OH ^−^_(s)_ (Figure 14C,E) (Equations (11)–(16)). All prepared catalytic systems can perform this step as resulted from experimental data These radicals represent the core of the process by performing the oxidation of paracetamol molecules and leading to complete mineralization of organic substrate (Equations (17) and (18)). As already stated, ammonia is a refractory to oxidation pollutant therefore, require significant amount of radical species depending on reaction conditions [89]. However it can be observed that catalytic systems are able to sustain several parallel processes and eliminating the toxic compounds like ammonia (Figure 14F). Mixed components thin films present some properties which can improve their catalytic activity. Hence in case of (Ti-Sn) system the photogenerated electrons from TiO_2_ conduction band migrate into the SnO_2_ conduction band while formed holes migrate backwards (Equations (19) and (20)) [90]. This is an important step in delaying the recombination of electrons and holes. In case of (Ti-Ce) system two significant phenomena occur: on CeO_2_ oxide surface some oxygen vacancies are formed through interaction with oxygen species from gas/liquid phase and a redox couple Ce^3+^/Ce^4+^ (Figure 14D) is formed [91,92]. Furthermore, Ce^3+^ is able to generate radicalic species in a similar manner with electrons from the conductive band (Equations (21) and (22)); a transfer of electrons from CeO_2_ also occursconduction band to TiO_2_ conduction band since bandgap energy of CeO_2_ is situated at more negative values (−0.37 eV) than those of TiO_2_ (−0.24 eV) while holes migrate in opposite directions (Equations (23) and (24)) [92]. Hence, (Ti-Ce-Sn) system apart from a slower recombination of charges may be subject to electron agglomeration in SnO_2_ conduction band and of holes in CeO_2_ valence band.

O_3_ direct reactions:

(6) $$O_{3}+Pa\;\to Oxidation\;by-products$$

(7) $$O_{3}\to O_{2}+O\;\left(1D\right)$$

O_3_/UV reactions:

(8) $$O_{3}+h\upsilon \;\left(254\;nm\right)\to O_{2}+O\;\left(1D\right)$$

(9) $$O\;\left(1D\right)+H_{2}O\to H_{2}O_{2}+2OH●$$

(10) OH●+Pa→Oxidation by−products

O_3_/UV/catalyst reactions:

(11) $$h\upsilon +TiO_{2}\to h^{+}+e^{-}\;$$

(12) $$OH_{\left(s\right)}^{-}+h^{+}\to OH●$$

(13) $$H_{2}O_{\left(s\right)}+h^{+}\to H^{+}+OH●$$

(14) $$O_{3}+e^{-}\to O_{3}^{-}●$$

(15) $$O_{3}^{-}●+H^{+}\to HO_{3}$$

(16) $$HO_{3}●\to OH●+O_{2}$$

(17) $$h^{+}/OH●/O\left(1D\right)+Pa\to Oxidation\;by-products$$

(18) $$e^{-}+Pa\to Reduction\;by-products$$

(19) $$TiO_{2}/SnO_{2}+h\upsilon \;\to TiO_{2}\left(h^{+}+e^{-}\right)/SnO_{2}\left(h^{+}+e^{-}\right)$$

(20) $$TiO_{2}\left(h^{+}+e^{-}\right)/SnO_{2}\left(h^{+}+e^{-}\right)\;\to TiO_{2}\left(h^{+}\right)/SnO_{2}\left(e^{-}\right)$$

(21) $$Ce^{4+}+e^{-}\to Ce^{3+}$$

(22) $$O_{3}+Ce^{3+}\;\to Ce^{4+}+O_{3}^{-}●$$

(23) $$TiO_{2}/CeO_{2}+h\upsilon \;\to TiO_{2}\left(h^{+}+e^{-}\right)/CeO_{2}\left(h^{+}+e^{-}\right)$$

(24) $$TiO_{2}\left(h^{+}+e^{-}\right)/CeO_{2}\left(h^{+}+e^{-}\right)\;\to TiO_{2}\left(e^{-}\right)/CeO_{2}\left(h^{+}\right)$$

## 4. Conclusions

For several years, catalytic ozonation represents an important alternative for water treatment. Therefore, intensive work was made in order to improve this process. In this study, paracetamol (a widely used NSAID) catalytic ozonation using TiO_2_-Me_x_O_y_ thin films was investigated. Using thin film in the oxidation removal of pollutants represents an important step for enhancement for this type of process and, in addition, to its simplification by avoiding the difficult step of catalyst separation. The present study describes for the first time in literature, the removal of paracetamol from aqueous solutions using thin films catalysts based on TiO_2_-Me_x_O_y_ nanosystems deposited on glass plates and immersed into a semi-batch reactor; the prepared thin films being very active in eliminating the targeted compound.

Considering that the initial concentration of the pollutant selected for this study is much higher compared with real-world effluents, the obtained results are very encouraging, giving the prospect of the complete elimination of the pollutants from aqueous effluents regardless of the compound type. The relative performances of noncatalytic processes can be attributed mainly to the presence of UV radiation since it is well-known that ozonation alone cannot cope with some resistance to oxidation chemical species. The higher than usual input concentrations represent a successful endurance test for the prepared catalytic systems.

## Figures and Tables

**Table 1:** The roughness of the prepared films.

Sample	RMS Roughness (nm)
(Ti)	1.1
(Ti-Ce)	5.9
(Ti-Sn)	2.97
(Ti-Ce-Sn)	2.1

**Table 2:** Band gaps determined from UV–Vis spectra.

Sample	Band Gap
(Ti)	3.68
(Ti-Ce)	3.44
(Ti-Sn)	3.68
(Ti-Ce-Sn)	3.38

**Table 3:** Ozone consumption during the oxidation process for prepared thin films in different conditions.

Catalytic System/Ozone Concentration	Consumed Ozone (mg)	Residual Ozone (mg)	Ozone Input (mg)	mgO_3_/mg TOC Removed (Consumed Ozone)	mgO_3_/mg TOC Removed (Ozone Input)
Glass, 12 O_3_	261.38	98.62	360	54.10	74.51
Ti, 4s, 12 O_3_	341.85	18.15	360	48.88	51.48
Ti-Ce, 4s, 12 O_3_	254.86	105.14	360	36.44	51.48
Ti-Sn, 4s, 12 O_3_	290.01	69.99	360	47.52	58.98
Ti-Ce-Sn, 4s, 12 O_3_	286.53	73.47	360	39.53	49.67
Ti, 2s, 12 O_3_	324.45	35.55	360	45.57	50.56
Ti-Ce, 2s, 12 O_3_	343.54	16.46	360	67.54	70.78
Ti-Sn, 2s, 12 O_3_	327.26	32.74	360	77.99	85.80
Ti-Ce-Sn, 2s, 12 O_3_	342.22	17.78	360	61.17	64.35
Glass, 6 O_3_	157.34	22.66	180	412.47	471.88
Ti, 2s, 6 O_3_	160.72	19.28	180	33.26	37.25
Ti-Ce, 2s, 6 O_3_	164.79	15.21	180	40.50	44.24
Ti-Sn, 2s, 6 O_3_	155.56	24.44	180	33.98	39.32
Ti-Ce-Sn, 2s, 6 O_3_	167.28	12.72	180	54.82	58.98

**Table 4:** Kinetic parameters for the paracetamol oxidation in different experimental conditions.

Catalytic System/Ozone Concentration	*k_obs_* (min^−1^) 10^2^	*R* ^2^
Glass, 12 O_3_	4.07	0.9913
Ti, 4s, 12 O_3_	6.12	0.98778
Ti-Ce, 4s, 12 O_3_	5.37	0.99401
Ti-Sn, 4s, 12 O_3_	6.26	0.99085
Ti-Ce-Sn, 4s, 12 O_3_	5.19	0.98929
Ti, 2s, 12 O_3_	3.90	0.98754
Ti-Ce, 2s, 12 O_3_	4.97	0.99081
Ti-Sn, 2s, 12 O_3_	4.51	0.98938
Ti-Ce-Sn, 2s, 12 O_3_	4.91	0.9672
Glass, 6 O_3_	0.23	0.99029
Ti, 2s, 6 O_3_	4.04	0.97132
Ti-Ce, 2s, 6 O_3_	4.35	0.95859
Ti-Sn, 2s, 6 O_3_	3.99	0.98008
Ti-Ce-Sn, 2s, 6 O_3_	2.87	0.98847

## Data Availability

All data are available upon reasonable request from the authors.

## Supplementary Materials

The following supporting information can be downloaded at: https://www.mdpi.com/article/10.3390/nano12040613/s1, Figure S1: FTIR spectra of prepared film before and after use in oxidation reaction; Figure S2: Evolution of paracetamol normalized concentration during the oxidation process for (Ti) system for two successive cycles at lower ozone concentrations and 2 number of slides; Figure S3: Evolution of paracetamol normalized concentration during the oxidation process for (Ti-Ce) system for two successive cycles at lower ozone concentrations and 2 number of slides; Figure S4: Evolution of paracetamol normalized concentration during the oxidation process for (Ti-Sn) system for two successive cycles at lower ozone concentrations and 2 number of slides; Figure S5: Evolution of paracetamol normalized concentration during the oxidation process for (Ti-Ce-Sn) system for two successive cycles at lower ozone concentrations and 2 number of slides; Figure S6: Evolution of normalized UV254 parameter during the oxidation process for (Ti) system for two successive cycles at lower ozone concentrations and 2 number of slides; Figure S7: Evolution of normalized UV254 parameter during the oxidation process for (Ti-Ce) system for two successive cycles at lower ozone concentrations and 2 number of slides; Figure S8: Evolution of normalized UV254 parameter during the oxidation process for (Ti-Ce) system for two successive cycles at lower ozone concentrations and 2 number of slides; Figure S9: Evolution of normalized UV254 parameter during the oxidation process for (Ti-Ce) system for two successive cycles at lower ozone concentrations and 2 number of slides.
