# Supplementary material for: Removal of Paracetamol from Aqueous Solutions by Photocatalytic Ozonation over TiO2-MexOy Thin Films
Source: Nanomaterials (Basel). 2022 Feb 11;12(4):613. doi: 10.3390/nano12040613 (PMC8875729; doi:10.3390/nano12040613)
Supplement: Supplementary file 1 [file nanomaterials-12-00613-s001.zip › nanomaterials-1560401-supplementary.pdf]

nanomaterials

Article

Removal of Paracetamol from Aqueous Solutions by Photocatalytic Ozonation over TiO_2-Me_xO_y Thin Films

Sorin Marius Avramescu [1,2], Irina Fierascu [3,4,*], Radu Claudiu Fierascu [3,5,*], Roxana Ioana Brazdis [3,5], Angel Vasile Nica [2], Claudia Butean [6], Elena Alina Olaru [2], Sorin Ulinici [7], Marian Nicolae Verziu [8] and Anca Dumitru [9,*]

1 Department of Organic Chemistry, Biochemistry and Catalysis, Faculty of Chemistry, University of Bucharest, 050663; Bucharest, Romania; sorin.avramescu@g.unibuc.ro
2 PROTMED Research Centre, University of Bucharest, 050107 Bucharest, Romania; nica.angelvasile@yahoo.ro (A.V.N.); alina.olaru@g.unibuc.ro (E.A.O.)
3 Emerging Nanotechnologies Group, National Institute for Research & Development in Chemistry and Petrochemistry—ICECHIM, 060021 Bucharest, Romania; roxana.brazdis@icechim.ro
4 Faculty of Horticulture, University of Agronomic Sciences and Veterinary Medicine of Bucharest, 011464 Bucharest, Romania
5 Department of Science and Engineering of Oxide Materials and Nanomaterials, Faculty of Chemical Engineering and Biotechnologies, University Politehnica of Bucharest, 011061 Bucharest, Romania
6 Department of Chemistry and Biology, North University Centre of Baia Mare, Technical University of Cluj-Napoca, 430122, Baia Mare, Romania; dee1168@yahoo.com
7 S.C. ICPE Bistrita S.A,420035 Bistrita, Romania; sorin_ulinici@icpebn.ro
8 Department of Bioresources and Polymer Science, Faculty of Applied Chemistry and Materials Science, University Politehnica of Bucharest, 011061 Bucharest, Romania; marian.verziu@upb.ro
9 Faculty of Physics, University of Bucharest, 077125, Măgurele Romania
* Correspondence: irina.fierascu@icechim.ro (I.F.); fierascu.radu@icechim.ro (R.C.F.); anka.dumitru@gmail.com (A.D.)

Citation: Avramescu, S.M.; Fierascu, I.; Fierascu, R.C.; Brazdis, R.I.; Nica, A.V.; Butean, C.; Olaru, E.A.; Ulinici, S.; Verziu, M.N.; Dumitru, A. Removal of Paracetamol from Aqueous Solutions by Photocatalytic Ozonation over TiO_2-Me_xO_y Thin Films. *Nanomaterials* **2022**, *12*, 613. https://doi.org/10.3390/nano12040613

Academic Editor: Baizeng Fang

Received: 31 December 2021
Accepted: 6 February 2022
Published: 11 February 2022

Publisher's Note: MDPI stays neutral with regard to jurisdictional claims in published maps and institutional affiliations.

Copyright: © 2022 by the authors. Licensee MDPI, Basel, Switzerland. This article is an open access article distributed under the terms and conditions of the Creative Commons Attribution (CC BY) license (https://creativecommons.org/licenses/by/4.0/).

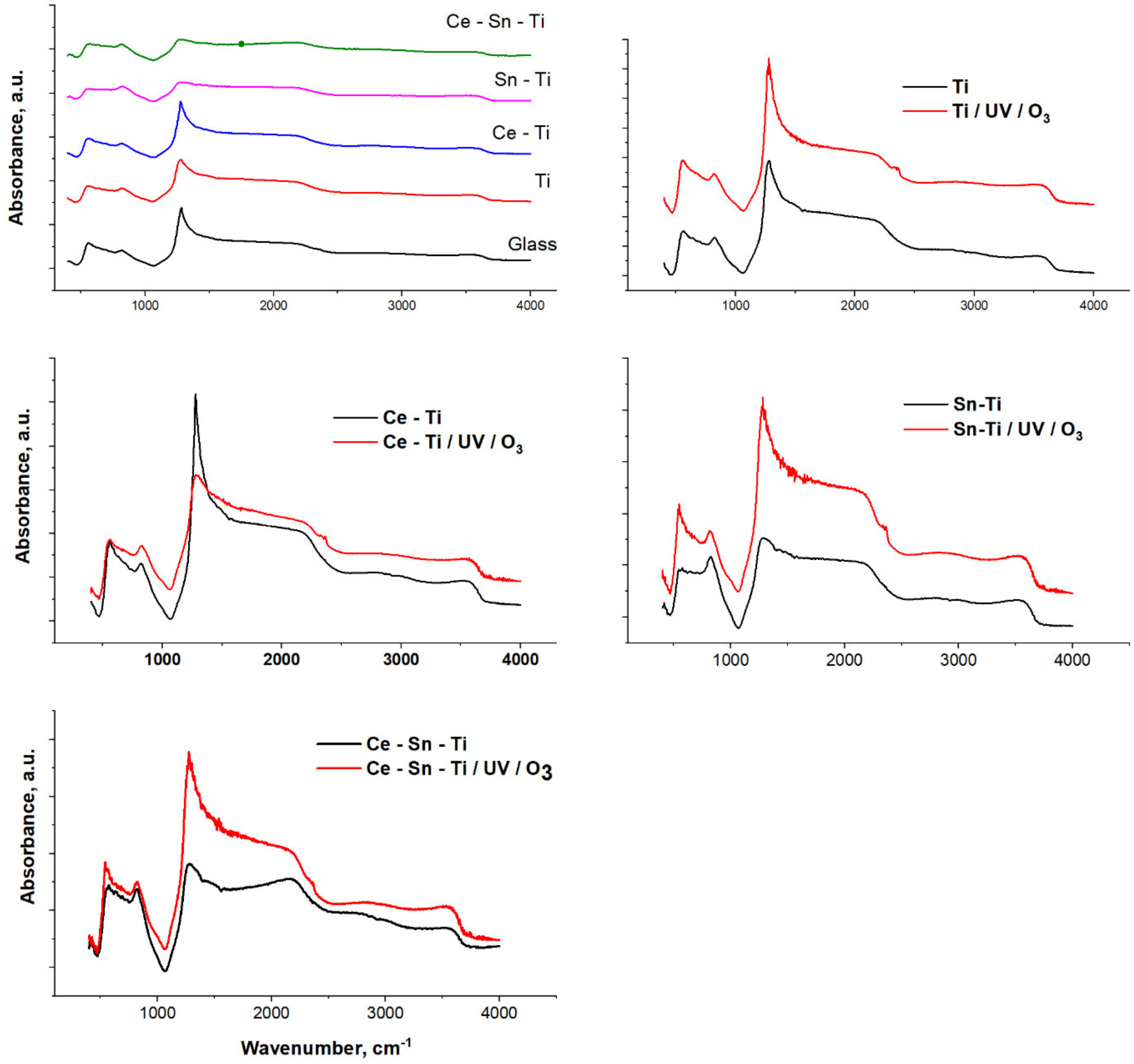

Figure S1. FTIR spectra of prepared film before and after use in oxidation reaction.

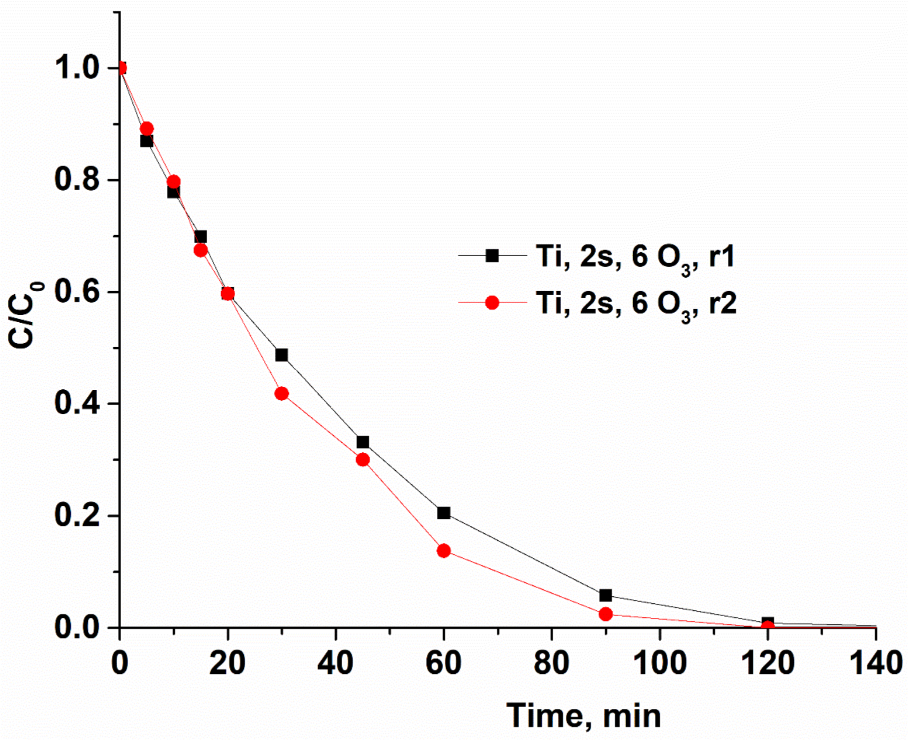

Figure S2.. Evolution of paracetamol normalized concentration during the oxidation process for (Ti) system for two successive cycles at lower ozone concentrations and 2 number of slides.

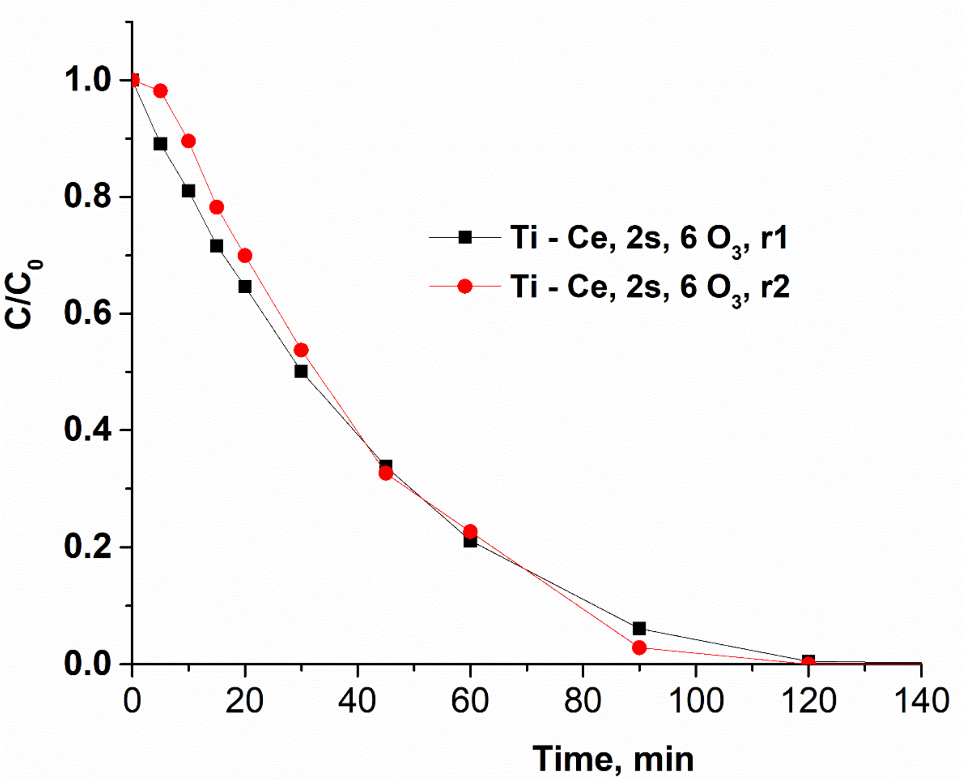

Figure S3. Evolution of paracetamol normalized concentration during the oxidation process for (Ti-Ce) system for two successive cycles at lower ozone concentrations and 2 number of slides.

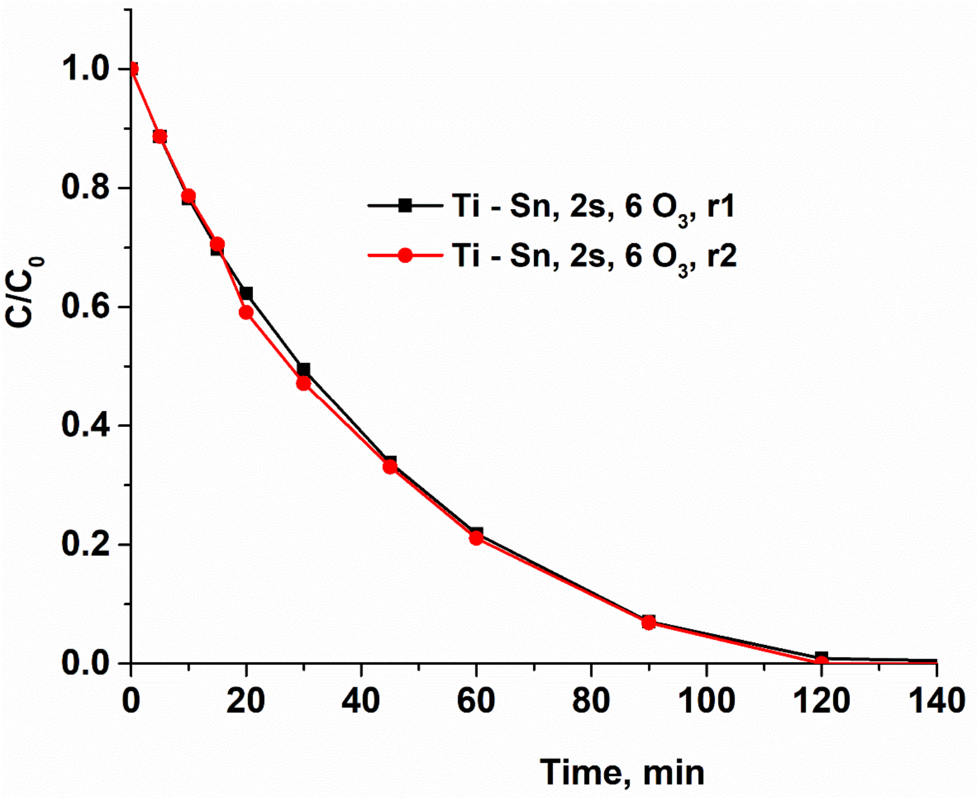

Figure S4. Evolution of paracetamol normalized concentration during the oxidation process for (Ti-Sn) system for two successive cycles at lower ozone concentrations and 2 number of slides.

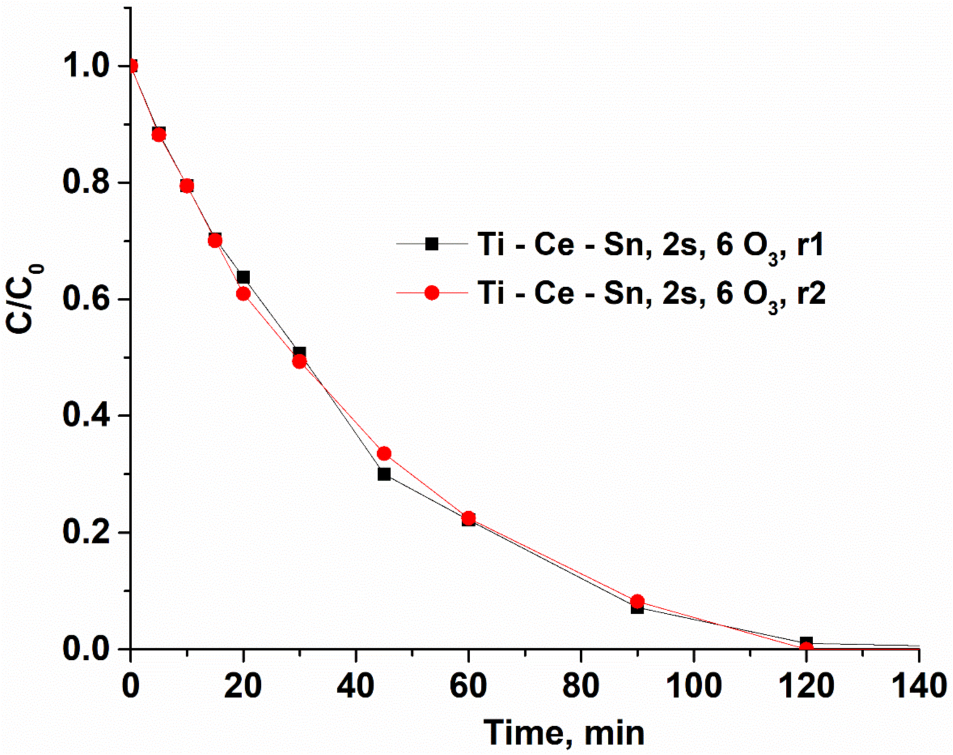

Figure S5. Evolution of paracetamol normalized concentration during the oxidation process for (Ti-Ce-Sn) system for two successive cycles at lower ozone concentrations and 2 number of slides.

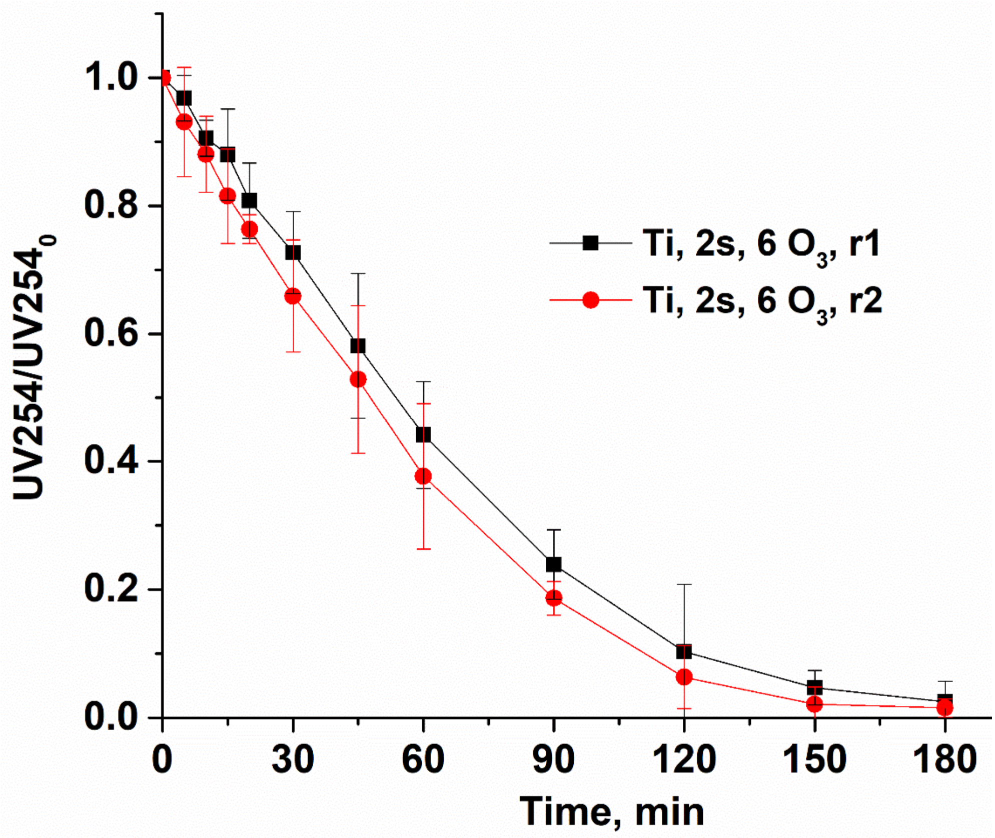

Figure S6. Evolution of normalized UV254 parameter during the oxidation process for (Ti) system for two successive cycles at lower ozone concentrations and 2 number of slides.

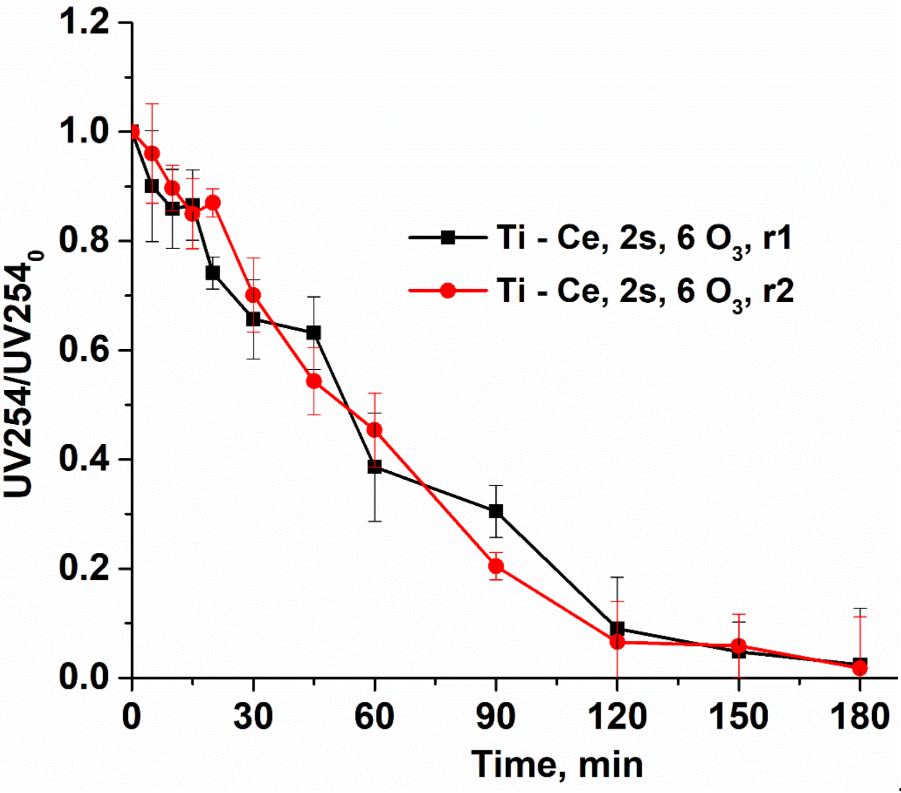

Figure S7. Evolution of normalized UV254 parameter during the oxidation process for (Ti-Ce) system for two successive cycles at lower ozone concentrations and 2 number of slides.

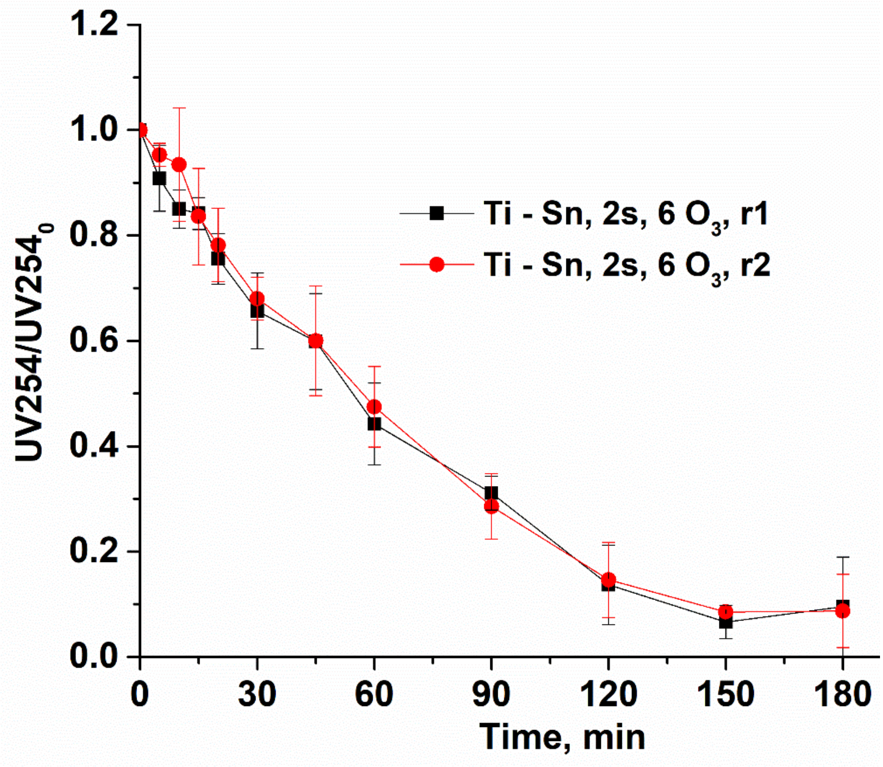

Figure S8. Evolution of normalized UV254 parameter during the oxidation process for (Ti-Ce) system for two successive cycles at lower ozone concentrations and 2 number of slides.

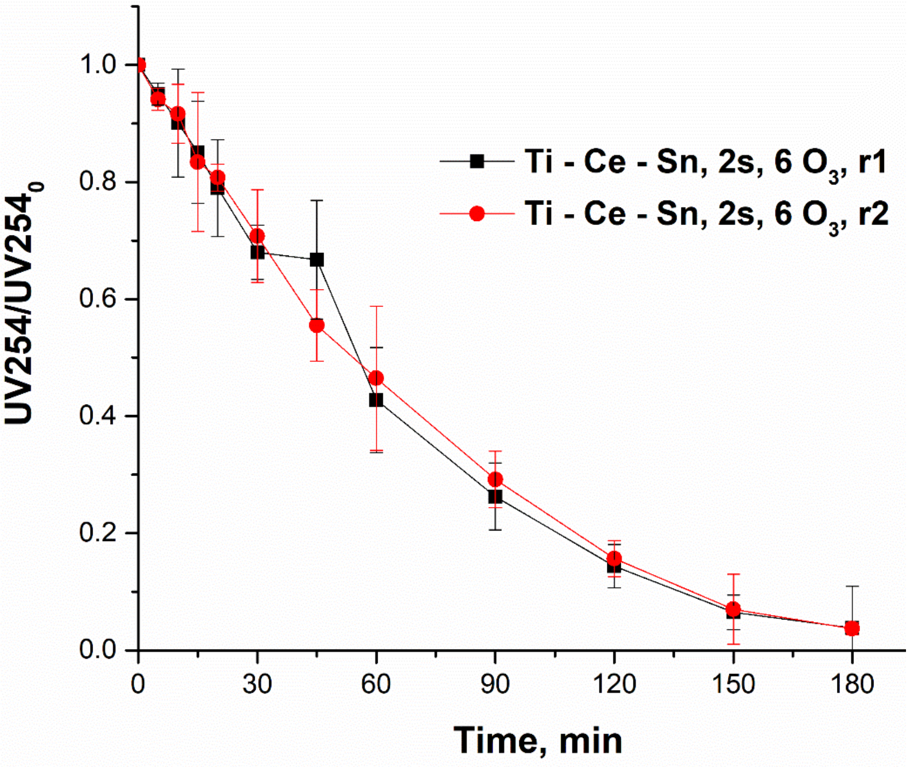

Figure S9 Evolution of normalized UV254 parameter during the oxidation process for (Ti-Ce) system for two successive cycles at lower ozone concentrations and 2 number of slides.
